# Surface potential and thin film quality of low work function metals on epitaxial graphene

**DOI:** 10.1038/s41598-018-34595-1

**Published:** 2018-11-07

**Authors:** Matthew DeJarld, Paul M. Campbell, Adam L. Friedman, Marc Currie, Rachael L. Myers-Ward, Anthony K. Boyd, Samantha G. Rosenberg, Shojan P. Pavunny, Kevin M. Daniels, D. K. Gaskill

**Affiliations:** 1 US Naval Research Laboratory, Washington, District of Columbia 20375 USA; 2 Laboratory for Physical Sciences, College Park, MD 20740 USA; 3 Department of Electrical and Computer Engineering, University of Maryland, College Park, MD 20742 USA

## Abstract

Metal films deposited on graphene are known to influence its electronic properties, but little is known about graphene’s interactions with very low work function rare earth metals. Here we report on the work functions of a wide range of metals deposited on n-type epitaxial graphene (EG) as measured by Kelvin Probe Force Microscopy (KPFM). We compare the behaviors of rare earth metals (Pr, Eu, Er, Yb, and Y) with commonly used noble metals (Cr, Cu, Rh, Ni, Au, and Pt). The rare earth films oxidize rapidly, and exhibit unique behaviors when on graphene. We find that the measured work function of the low work function group is consistently higher than predicted, unlike the noble metals, which is likely due to rapid oxidation during measurement. Some of the low work function metals interact with graphene; for example, Eu exhibits bonding anomalies along the metal-graphene perimeter. We observe no correlation between metal work function and photovoltage, implying the metal-graphene interface properties are a more determinant factor. Yb emerges as the best choice for future applications requiring a low-work function electrical contact on graphene. Yb films have the strongest photovoltage response and maintains a relatively low surface roughness, ~5 nm, despite sensitivity to oxidation.

## Introduction

Graphene, a single atomic layer of sp^2^ bonded carbon atoms arranged in a hexagonal lattice, has remarkable photonic properties, absorbing approximately 2.3% of incoming light in the infrared to ultraviolet range^1–3^. These photonic properties, when considered in combination with graphene’s high Fermi velocity, high mobility, and relativistic quantum electronic transport, suggest the opportunity to create a variety of unique photonic devices^4–9^. Among these devices, graphene optical detectors have been demonstrated with light intensity modulations of 40 GHz without degradation of the photoresponse, and transfer data as fast as 12 Gbits s^−1^ ^10–12^. Such devices have great potential for use in the development of faster data transfer networks for optical telecommunications. Graphene optoelectronic devices operating in the terahertz gap between 0.1 and 10 THz are even more promising^13–16^, as THz technology in this range is emerging as a potential solution for a variety of problems in fields as diverse as cancer detection, atmospheric monitoring, and security screening^17–19^. Graphene’s two-dimensional lattice affords limited opportunities for lattice-electron energy loss^20–22^, thereby causing absorbed THz radiation to induce a hot-carrier effect, a useful mechanism for THz detection^20–22^. Graphene-based THz detectors currently achieve responsivities as high as 400 V W^−1^ and there are good indications for potential improvement^18,19,23^.

While conventional semiconducting photodiodes utilize a P-N junction, efficient graphene devices can be fabricated with a single strip using different metals for the source and drain contacts^11,23^. Metal films can dope graphene, causing junctions to form at the edge of each contact, thus generating a photoresponse when illuminated^24^. Scanning photocurrent measurements across metal-graphene-metal junctions reveal that the photoresponse is maximized near each metal-graphene junction, but vanishes in the center of the channel^21,25,26^. The photoresponse is dependent upon a combination of photovoltaic effects from the induced electric field and photothermoelectric effects from the Seebeck coefficient differences of differently doped graphene^11,27–29^. The mechanism is similar to the hot-carrier dominated photoresponse at graphene P-N junctions^28^. Moreover, the amount of doping induced by the metal is largely determined by its work function, *ϕ*^24^. This is important in detectors because using different metals at the source and drain creates an asymmetry that induces a net photoresponse, with the magnitude determined by the *Δϕ* of the two metals; this means that the maximum response should be obtained using contacts with the largest work function difference. Photodetectors operating in both the telecommunication and THz spectrum have already been developed using this type of architecture^11,23^. While back gating can improve performance, passive THz devices have responsivities as high as 200 V W^−1^ ^23^. This asymmetric contact design enables rapid and wafer-scale fabrication of graphene devices by minimizing processing steps and reducing the need for split gating.

Despite the usefulness of metal-graphene junctions, little published work exists that quantifies the behavior of different work function metals on graphene that can aid the optimization of device design and fabrication. While there has been extensive work on graphene-metal contacts, these studies largely pertained to achieving low contact resistance and have focused on metals with high work functions^11,23,26,28,30–35^. Therefore, a preliminary study of the qualitative interactions between graphene and metals with a large range of *ϕ* is essential for various applications including photodetectors utilizing metal contacts that induce a voltaic asymmetry. There is very limited research on low work function thin films on graphene. The majority of graphene studies involving erbium and ytterbium metals use the metals as dopants in laser fibers^36,37^, while europium oxide films on graphene are used for spintronics due to EuO’s ferromagnetic capabilities^38–40^, and yttrium oxide is used as a high κ dielectric^41,42^. However, to our knowledge there are no studies in the literature that characterize these metals or their oxides for their capability to function as an electrical source/drain contact. For example, pairing a low work function contact with a noble metal should result in improved performance of graphene-based two-terminal photodetectors.

In this study, we deposit metals with work functions ranging between 2.5 eV and 5.8 eV on graphene grown epitaxially on SiC via Si sublimation. We find a discrepancy between the reported and measured values of *ϕ* for the low work function category, with the measured *ϕ* consistently higher than expected. A number of metals introduce significant processing challenges and unique characteristics when deposited on graphene. Er and Pr films form micrometer-sized topographic features likely due to adhesion issues and complications from volumetric changes during rapid oxidation. Eu films exhibit unique phonon signatures when on graphene. A thorough examination of the graphene-metal interface at the Er film edge shows evidence of disorder in the graphene lattice, suggesting that sp^3^ bonding or oxidation of the graphene lattice may occur during metal oxidation. For Eu deposition, we also report on a redshift in the graphene Raman spectrum, possibly due to Eu-adsorption or Eu intercalation with the graphene lattice^4^. A preliminary analysis of photoresponse uncovers no correlation between photovoltage and surface potential, with the photovoltage values largely determined by the physical characteristics of the metal-graphene interface. Of the rare earth films studied, Yb films are the most encouraging because they exhibit the strongest photoresponse and smallest surface roughness.

## Results and Discussion

**Figure 1:**
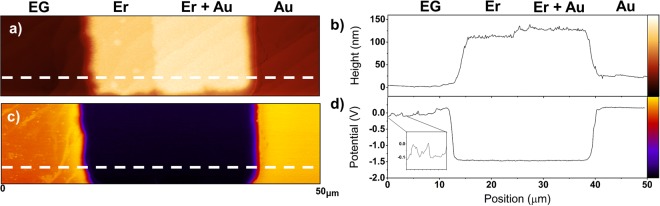
(**a**) AFM and (**c**) KPFM scans over a lateral range of 50 μm overlapping Er and Au pads on EG and (**b**,**d**) are the corresponding line scans taken from the indicated dotted region. The 100 nm and 25 nm heights of the Er and Au pad are clear in the AFM line scan, with a 25 nm jump at the point of overlap. Such distinctions are nonexistent in the KPFM line profile (**d**) as the surface potential is determined by only a few layers near the surface. The potential difference between 1 ML graphene (located at the terraces) and 2 ML graphene (located at the steps) is extracted from the graphene portion of the KPFM scan (from 0 to 12 μm) and is 0.11 eV. The bumps in this portion of the KPFM line profile corresponds to the surface potential change due to 2 ML of graphene.

Figure 1 shows examples of AFM and KPFM scans used for the metals in this study. Figure 1a is an AFM scan of EG with an Er metal pad overlapping a Au metal pad. The region of the Er pad that overlaps the Au is clearly visible in the center of the scan, due to the increased height (Fig. 1b). From the line profile (Fig. 1b), the height of the Au and Er pads are 25 nm and 100 nm, respectively, and are consistent with the metal evaporation conditions for this sample. The step edges of the SiC substrate in both the uncovered EG and underneath the metal pads are observed to run diagonally from the bottom left to the top right of the image. Figure 1c is a KPFM image of the same scan area measuring the relative surface potential, with Er and Au having low and high potentials, respectively. From the line profile (Fig. 1d), the potential difference between the Er pads and the Au pads and graphene is −1.39 ± 0.04 eV and +0.33 ± 0.04 eV, respectively. On the left side of Fig. 1c, the bright diagonal lines correspond to the step edges. The corresponding troughs and peaks in the surface potential of the graphene side in Fig. 1d correspond to differences in the graphene potential between terraces and step edges. We measure this difference to be 0.11 ± 0.04 eV, with a higher potential for the graphene along the step edges where 2 or 3 monolayers (ML) of graphene are present (inset in Fig. 1d). This value is consistent with other reports which measure the difference as 0.135 ± 0.009 eV and 0.11 ± 0.02 eV^43,44^.

We note here an important characteristic of KPFM scans. For the metal thicknesses used, KPFM measurements are not sensitive to sample (deposited metal) topography and, in the absence of chemical reactions, only depend on the material properties near the surface. This characteristic is exemplified by the Er scans. The interface where the Er pad overlaps the Au pad is not visible in KPFM (Fig. 1d). Yet, the SiC step edges underneath the metals are readily seen in the AFM image (Fig. 1a). We performed an additional test of this characteristic by depositing Eu, Y, Pt, and Ni on a gold-coated n-type Si wafer. The measured surface potential differences (SPD) of these metals with respect to Au on Si were identical to those measured on EG/SiC substrates. The only exception to this characteristic is in the case of Y and Cr films, as these react to form another compound; this is discussed below.

A summary of the reported (*ϕ*_*R*_) and calculated work functions (*ϕ*_*M*_) of the metals deposited in this study, as well as the measured SPD between the metal film and graphene (SPD_M-G_) is found in Table 1^45,46^. The measured values and variance presented in Table 1 are found from averaging 5–7 different pads for each corresponding metal and their associated interfaces. We assume the work function for polycrystalline Au, *ϕ*_*Au*_, is 5.10 eV based on referenced values of Au and the SPD between Au and graphene^46–48^. We use this value as our comparative reference to calculate the work functions for graphene and the other metals using the measured surface potential. For example, to calculate the work function of EG on a terrace using data found in Fig. 1c, we compare the SPD between graphene and gold. We measure the SPD directly from the KPFM measurement values and then use the charge of an electron (*q*) and the value of *ϕ*_*Au*_ *to find* *ϕ*_*G*_.

1 $$SP{D}_{G-Au}=KPF{M}_{G}-KPF{M}_{Au}$$

2 $${{\Phi }}_{G}=\frac{{{\Phi }}_{Au}+SP{D}_{G-Au}}{q}$$

**Table 1:** KPFM Surface Potential and Photovoltage Measurements on Graphene with various deposited metals using a Au standard.

Metal	Reported *Φ*_*R*_ (eV)	*SPD*_*M-G*_ (V)	*Φ*_*C*_ (eV)	RMS Surface Roughness (nm)
Europium	2.50^45,46^	−1.03 ± 0.02	3.74 ± 0.06	36 ± 5
Ytterbium	2.60^45^	−1.13 ± 0.02	3.64 ± 0.06	5.0 ± 0.3
Praseodymium	2.70^46^	+0.10–0.54* ± 0.03	4.87–5.31* ± 0.07	140 ± 20
Yttrium	3.1^47,48^	−1.50 ± 0.05	3.27 ± 0.09	2.7 ± 0.2
Erbium	3.12^45^	−1.53–1.39* ± 0.04	3.24–3.38* ± 0.08	60 ± 10
Chromium	4.5^47,48^	+0.33 ± 0.01	5.10 ± 0.05	1.4 ± 0.3
Copper	4.65^47,48^	+0.36 ± 0.05	5.13 ± 0.09	1.6 ± 0.3
Rhodium	4.98^47,48^	+0.38 ± 0.03	5.15 ± 0.07	3.3 ± 0.6
Nickel	5.15^47,48^	+0.02 ± 0.04	4.79 ± 0.08	1.6 ± 0.2
Gold	5.10^46–48^	+0.33 ± 0.04	5.10	1.3 ± 0.1
Platinum	5.65^47,48^	+0.41 ± 0.04	5.18 ± 0.08	1.5 ± 0.3

Measurements were done in the laboratory ambient at room temperature and the rare earth metals may be partially oxidized which approximates real world conditions. The value of the work function of graphene was calculated to be Φ_G_ = 4.77 ± 0.04 eV. The various headings in the table are defined in the text below. The pre-metal deposition roughness of graphene was measured as 1.7 ± 0.2 nm. ^*^SPD changed over time. The asterisked value corresponds to the SPD_M-G_ at t = 60 minutes.

We obtain *ϕ*_*G*_ = 4.77 ± 0.04 eV. We find this value of *ϕ*_*G*_ is the same for all samples used in this work. Furthermore, the value is consistent with other reports for 1 ML EG^49–51^. For the deposited metals, the calculated work function values, *ϕ*_*C*_, in Table 1 are calculated from Eq. 3 using *ϕ*_*G*_ = 4.77 ± 0.04 eV.

3 $${{\Phi }}_{M}=\frac{{{\Phi }}_{G}+SP{D}_{M-G}}{q}$$

**Figure 2:**
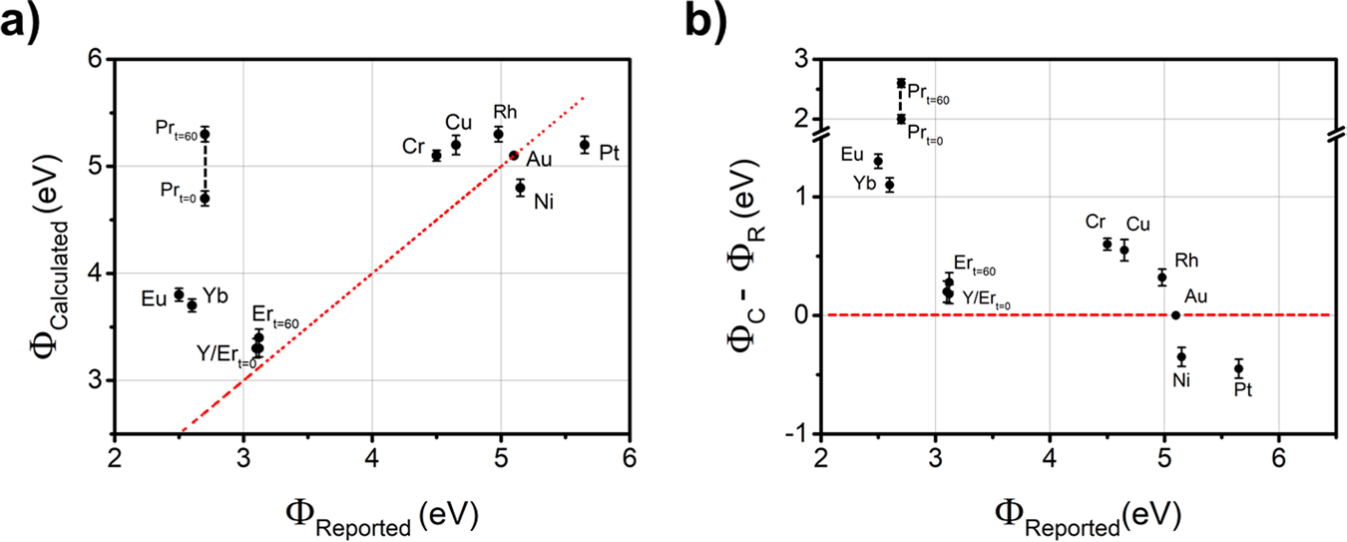
(**a**) Calculated work function values of metals on EG and b) the difference between calculated and reported work function values. A red dotted line indicates Φ_C_ = Φ_R._ The low work function metals show consistently higher measured values than the values reported in the literature. Pr and Er Φ change with time with Pr/Er_t=0_ and Pr/Er_t=60_ referring to immediately after deposition and 60 minutes after deposition, respectively. In the case of Pr, there may be some capacitive charging or a strong polar surface associated with the higher value; see text for details.

Figure 2a shows a plot of *ϕ*_*C*_ vs. *ϕ*_*R*_, while Fig. 2b shows the difference *ϕ*_*C*_ − *ϕ*_*R*_ vs. *ϕ*_*R*_. From Fig. 2a, the high work function metals have measured values above and below the *ϕ*_*C*_ = *ϕ*_*R*_ line, whereas the low work function metals are consistently measured at values greater than what is reported previously and are spread over a wider range. The metals with the lowest and highest work functions reported in the literature are Eu and Pt with values 2.50 eV and 5.65 eV, respectively. However, the metals with the lowest and highest surface potentials measured here are Y and Pr with 3.27 ± 0.09 eV and 5.31 ± 0.07 eV, respectively. The measured values of Er and Pr changed over time. Immediately after removal from the vacuum chamber at t = 0 min, we measured SPD_Er-G_ and SPD_Pr-G_ to be −1.53 ± 0.04 eV and +0.10 ± 0.03 eV, respectively. An hour later at t = 60 min we measured SPD_Er-G_ and SPD_Pr-G_ to be −1.39 ± 0.04 eV and +0.54 ± 0.03 eV, respectively. Both Er and Pr will be discussed in depth later in the manuscript.

For the high work function metals, the discrepancy from literature values cannot be due to substrate effects since, as we discussed earlier, KPFM is sensitive to the surface properties of the metal; we also specifically tested and confirmed this characteristic using a different substrate. Moreover, the KPFM system is likely not the source of the discrepancy since we obtain reproducible results over the course of months and the calculated work function of graphene is the same as other reports that use calibrated KPFM tips^43,44^. In contrast, our metal evaporation system uses metals of less than ultra-high purity, operates at intermediate pressures, and is used by a wide variety of personnel. There are also unknown contaminants that are present in the vacuum chamber, including carbon, which may become incorporated into the film during deposition. These contaminants can change surface potential and may help to explain the values in Fig. 2. Hence, the discrepancy may be due to the deposited metal purity as it is well known that surface potential results can vary with purity^47,52^. In addition, surface potential values are also strongly dependent on surface orientation, which was not controlled in this work. For example, polycrystalline Au is reported as having a work function of 5.1 eV, whereas Au(100), Au(110), and Au(111) have work functions of 5.47, 5.31, 5.37 eV, respectively^47^. Similarly, the work function of Cu varies with orientation, ranging from 4.48–4.98 eV^47^. In any case, we emphasize that our KPFM results for the high work function metals are reproducible over the course of months.

The discrepancy from literature values for the low work function metals, in addition to the purity issues noted above, is likely due to oxidation. These metals are known to oxidize rapidly when exposed to the atmosphere. Despite measuring the surface potential directly after metal deposition, the metals Eu, Y, Er, Yb, and Pr, begin oxidizing upon removal from the deposition chamber. The oxidation is optically apparent on the samples because the appearances of the deposited rare earth metal films change from reflective, to opaque, and then to semi-transparent or transparent within minutes to hours. We confirmed the oxidation by XPS measurements; for example, XPS showed that the Yb (4d_5/2_) peak was located at 185.87 eV indicative of Ytterbium oxide. Because of the metal oxidation, the surface potential measurements are expected to differ from the pure metals. For the case of Pr, the calculated *ϕ* is expected to increase with increasing oxygen exposure, due to the presence of Pr_2_O_3_^53^, and this appears to be consistent with KPFM measurements in Fig. 2a. However, for Eu and Y, oxidation reduces the work function, with EuO and Y_2_O_3_ having reported *ϕ* values of 1.8 and 2.0 eV, respectively^54–56^. And the case of EuO is further complicated because the work function is strongly dependent on orientation, e.g., the work function of EuO(111) is 7.5 eV, which is considerably higher than 1.8 eV of EuO(100)^54^. In general, oxidation of the deposited metal will change *ϕ* and the data imply that it generally increases. The experimental conditions used in this work are similar to those encountered in processing actual devices, including exposure of the devices to ambient during the device fabrication and subsequent possibility of oxidation as exhibited here.

Since the KPFM measurements are of oxidized or partially oxidized surfaces, the measured values may vary from the work function of the pure metal. Nevertheless, we believe the values in Table 1 are still a useful indicator of the relative surface potential at the metal-graphene interface. Studies have shown that oxidation of metals at interfaces begins within the deposition chamber unless utilizing ultra-high vacuum deposition conditions^57^. Therefore, even though the Table 1 values are likely from a partially oxidized surface, the graphene-metal interface is also partially oxidized based on our chamber pressure of 1–3 × 10^−6^ Torr.

**Figure 3:**
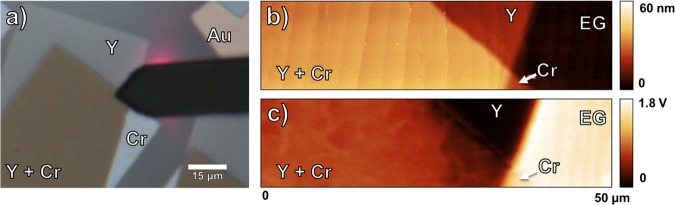
(**a**) Optical image during AFM scan, (**b**) AFM height scan, and (**c**) KPFM scan of Y and Cr pads on EG. The Y + Cr regions have a unique structure evident from the KPFM measurements, likely due to the formation of Y_2_CrO_3_.

To help guide utilization of the five rare earth metals for graphene applications, we look more closely at the film characteristics after deposition on EG. Y films were deposited after Cr causing Y to overlap Cr, and one such intersection is shown in Fig. 3. The optical image was taken during surface probe scanning and includes the AFM cantilever (Fig. 3a). The Y pad is transparent, making borders of the underlying Cr pad visible. Morphology issues of the Y films occurred over a time period of months and are discussed in detail below. Four different regions are seen in the AFM height scan (Fig. 3b), with the Y and Cr pads having heights of 24 and 28 nm, respectively. In the KPFM scan (Fig. 3c), the EG, Y, and Cr regions have surface potentials that correspond to Table 1. However, the region where Y overlaps Cr (Y + Cr) has a calculated work function of 3.93 ± 0.09 eV (SPD_M-G_ = −0.84 ± 0.05 eV). Note that the Y work function does not change when overlapping gold, as is expected. In Fig. 3c, filamentary contrast lines are visible in the Y + Cr region and suggest that structure exists within the film having regions of differing potentials that vary by 0.11 eV. We believe the structure observed in the overlapping region in Fig. 3c is the result of Y and Cr reacting since the alloy Y_2_CrO_3_ has been reported to form during joint sputtering of Y and Cr films^58^. In that case, the overlapping region in Fig. 3c consists of Y_2_CrO_3_ mixed with Y and Cr and we propose 3.93 ± 0.09 eV as the mixture’s previously unreported work function. Of the eleven metals investigated in this work, this is the only instance of a metal’s work function changing when overlapping another metal.

**Figure 4:**
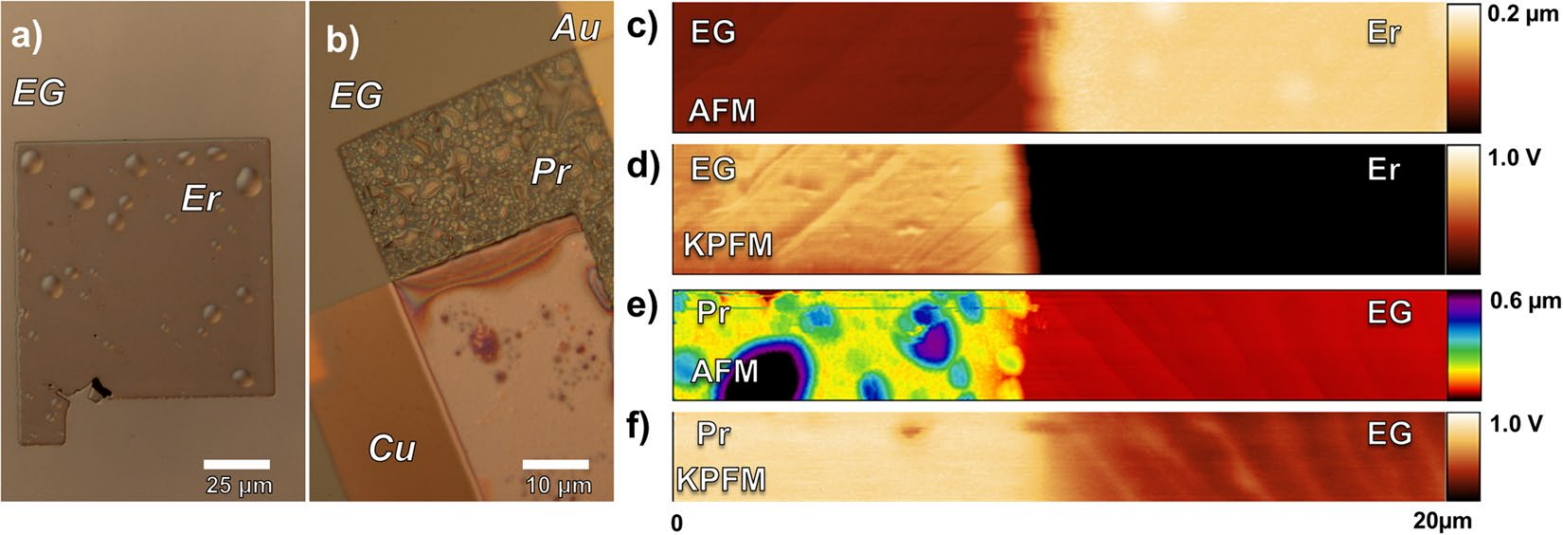
Optical images (**a**,**b**), AFM height scans (**c**,**e**) and KPFM scans (**d**,**f**) of Er (**a**,**c**,**d**) and Pr (**b**,**e**,**f**). Both metal films have large irregularities (bubbles) visible by optical microscopy and AFM images. Despite having surface features several hundreds of nanometers in height, the surface potential remains constant.

Er and Pr deposited films on EG have similar morphology and surface potential characteristics. Deposition of both metals (100 nm) on EG results in raised hemispherical features (bubbles) (Fig. 4). These bubbles are not immediately observed after removal from the deposition chamber, yet become apparent during KPFM measurements 1–3 hours later. Figure 4a is an optical image of an Er pad on EG; AFM scans, Fig. 4c, indicates the features range in height from 100 nm to greater than 5 μm. The features on Pr pads, shown optically in Fig. 4b, have a narrower AFM height range, Fig. 4e, from 500 nm to 1 μm. We use a spectrum color scale for the height map of the Pr films in order to see both the bubbles and the step edges of the substrate. These bubbles on EG result in significant roughness, 60 ± 10 and 140 ± 20 nm (RMS) for Er and Pr, respectively. Rather similarly, some bubble formation for Pr on the Cu pad is also evident, as shown in Fig. 4b where the two-toned color is due to the Pr metal overlapping part of the pad. However, these bubbles do not form when the metals are deposited on Au; in this case, the surface roughness is 5 ± 1 and 7 ± 2 nm (RMS) for Er and Pr, respectively, yet this is still rougher than the starting EG surface (1.7 ± 0.2 nm).

Since the bubble morphology evolves with exposure to the ambient atmosphere over a time span of 1–3 hours, this characteristic is likely due to oxidation of the Er and Pr films. This mechanism is supported by KPFM measurements, summarized in Table 1, demonstrating *ϕ* changes over time. Moreover, as noted earlier for the Pr deposited films, the magnitude of *ϕ* is consistent with the formation, at least in part, of Pr_2_O_3_. Figures 4d and 4f show KPFM scans of the Er and Pr deposited films. In both Figs. 4d and 4f, the step edges of the bare graphene are easily seen yet the films have a uniform potential, with no indication of raised features. Since the surface potential does not change over the entire deposited area, this implies that the entire region is chemically identical. We propose that, as the deposited metals oxidize, the films are likely to de-adhere from the EG surface, which lacks available bonding states, due to volumetric differences between the metal and metal oxide. For the case of films deposited on Cu and Au, available bonding states increase adherence and thus reduce bubble formation.

For the Pr deposited films, *ϕ*_Pr_ has an unusual dependence on the underlying material; it is the only metal whose surface potential is dependent on whether or not the scan is taken with respect to Au or Graphene. From Table 1, SPD_Pr-G_ is +0.10 ± 0.03 eV at t = 0 and +0.54 ± 0.03 eV at t = 60 min. However, when measured against Au the SPD_Pr-Au_ is −0.76 ± 0.02 eV at t = 0 and −0.48 ± 0.02 eV at t = 60 min. Using *ϕ*_*Au*_ = 5.10 eV, this corresponds to a *ϕ*_*Pr*_ of 4.34 ± 0.02 eV and 4.62 ± 0.02 eV at t = 0 and t = 60 respectively. This is a 0.53–0.69 ± 0.06 eV difference than when measured against graphene. It is unclear why this discrepancy occurs with Pr, but one possibility might be that the Pr is charging during measurement. Pr_2_O_3_ is known to be a high-κ dielectric oxide, and the graphene-Pr interface is likely very inconsistent considering the high surface roughness and tall features^59^. Alternatively, the Pr film is much smoother when overlapping Au, which perhaps discourages charging, resulting in a lower measured surface potential.

**Figure 5:**
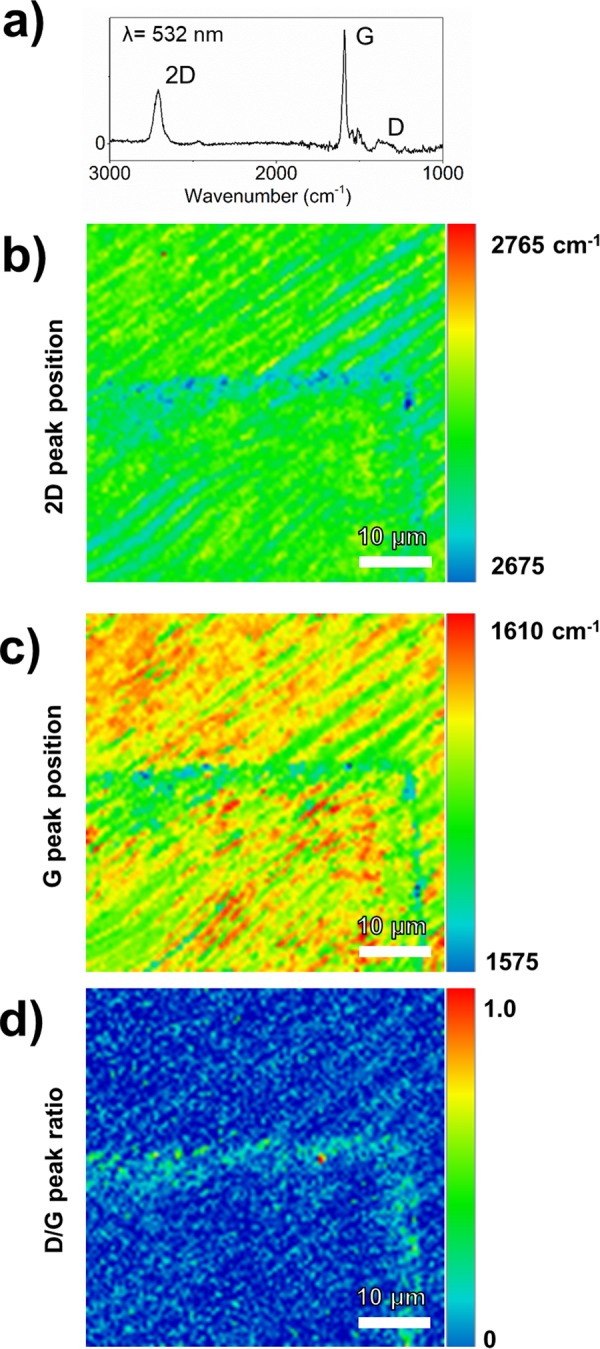
(**a**) Raman spectrum of Er deposition on EG after sonication to remove the deposition showing the 2D, G, and D peaks with the SiC spectrum subtracted. Post-sonication Raman spectroscopic map showing the graphene (**a**) 2D peak location (**b**) G peak location, and (**c**) D/G peak ratio. Distortion of the vibrational modes of graphene at the outline of the metal pad suggests graphene-metal oxide bonding.

To evaluate the effect of Er metal deposition on the underlying EG, including areas underneath the hemispherical features, the sample was sonicated in de-ionized water for 5 seconds. This removed the Er pads but not the Au pads, consistent with the hypothesis of poor adhesion. The exposed region was probed by Raman spectroscopy. Figure 5 shows a single point Raman spectrum and three Raman spectroscopic maps of regions where the Er pad had been removed via sonication. Figure 5a shows the Raman spectrum of EG with the SiC peaks subtracted. The 2D and G peaks, characteristic of graphene, are readily seen at 2710 and 1590 cm^−1^, respectively. The other peaks are from incompletely subtracted contributions of the SiC substrate. In the Raman maps, Fig. 5b shows the 2D peak position, Fig. 5c shows the G peak position, and Fig. 5d shows the ratio of D/G peak ratio. An imprint of the corner of the metal pad is visible in all three Raman scans. The majority of the EG that was once covered by the deposited Er is spectroscopically identical to areas that were not covered, including those that had bubbles; this provides further support for the previously discussed de-adhesion hypothesis. However, EG along the perimeter of the deposited metal shows a shift in the 2D peak, G peak, and increase in the D/G peak ratio. In Fig. 5b, the 2D peak shifts from 2710 ± 1 cm^−1^ in the unaffected graphene to 2685 ± 5 cm^−1^ along the perimeter. Likewise, the G peak in Fig. 5c shifts from 1600 ± 1 cm^−1^ to 1585 ± 5 cm^−1^. Spectroscopic shifts in these peaks are consistent with EG lattice strain and/or doping^60^. From Fig. 5d, the perimeter exhibits a D/G ratio of ~0.12 and 0.03 elsewhere. Since a low D/G peak ratio is often used as an indicator of high graphene film quality, the enhanced ratio at the perimeter indicates defects that break sp^2^ symmetry^60–62^. We propose that during oxidation of the Er pad the EG along the perimeter of the film is affected, reacting with the metal and/or oxide resulting in the formation of erbium carbide or forming graphene oxide. Furthermore, the Raman shifts in the G and 2D peaks are the result of strain created by the reactions at the perimeter^62^.

Another deposited metal that shows evidence of altering EG’s Raman signature is Eu. Figure 4 shows AFM, KPFM, optical, and Raman scans of Eu metal deposited on EG. When deposited on EG, the Eu forms a relatively rough surface with an RMS roughness of 36 ± 5 nm over graphene, (see from Table 1). Narrow features as high as 300 nm with faceted sides are visible in the AFM scan in Fig. 4a. When deposited over other metals, such as Au, the Eu forms a smooth continuous film with an RMS roughness of 2 nm and a height of 60 nm. Despite its rough surface on graphene, the potential in Fig. 4b remains relatively constant at 1.03 eV below that of graphene. In the image taken with an optical microscope in Fig. 4c, the Eu film deposited on Au is continuous in color and semi-transparent, causing the Au to appear darker in color. This semi-transparent appearance of the Eu film is similar to the appearance of the other rare earth metal films in this study. However, the Eu film deposited over graphene has a distinct blue color. Optically the film has a rough texture, which is in agreement with the AFM measurements. Bright white crystalline spots (size ~1 μm) appear randomly across the film, shown in small squares in Fig. 4c. For comparison, commercial powdered Europium oxide has a soft white color.

**Figure 6:**
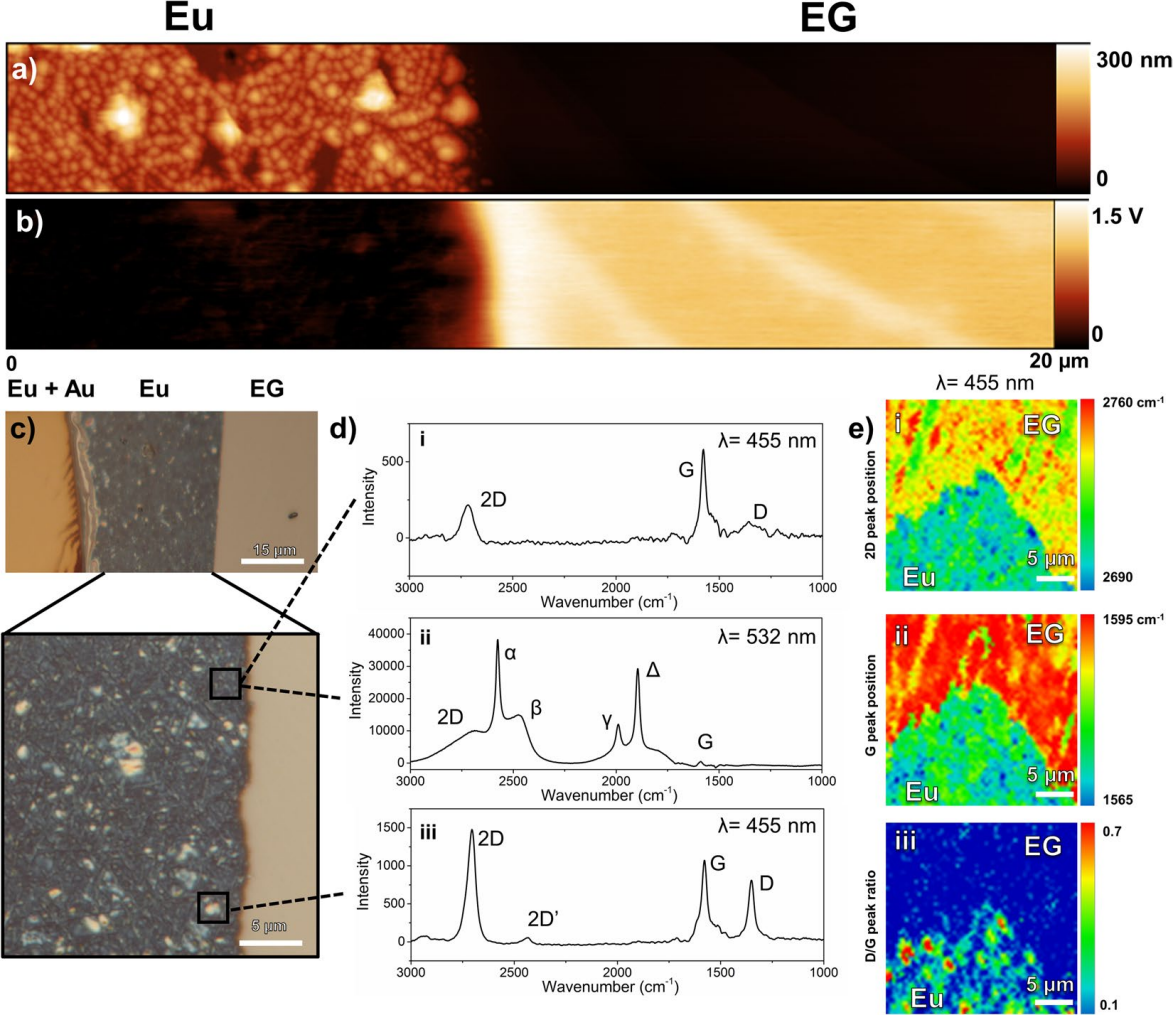
(**a**) AFM height and (**b**) KPFM potential scans of Eu on EG. (**c**) An optical image of the Eu film overlapping Au and graphene, including a higher magnification region of the Eu-graphene interface. (**d**) Raman spectra at different regions of the Eu film, denoted by the dashed lines, using (i,iii) 455 nm and (ii) 532 nm probes. Strong sharp peaks are observed in the Eu Raman spectrum, with intensities 10x that of the graphene-only spectrum. For these spectra, the SiC contributions have been subtracted. (**e**) Raman spectroscopic maps using a 455 nm laser showing the (i) 2D peak position (ii) G peak position, and (iii) D/G peak ratio.

We used Raman spectroscopy to characterize the EG covered by Eu. Figure 6d-i shows a Raman spectrum taken at a point in the blue region of the film with a 455 nm excitation. The Raman signature of EG underneath is easily measured through the deposited film showing the characteristic 2D and G peaks. The Raman measurements have a strong background fluorescence obscuring the underlying signatures; this result differs in comparison to all other deposited films. However, when the Raman spectra at the same point is measured using 532 nm laser, we observe a significantly different result, as seen in Fig. 6d-ii. The 2D and G peaks are still observable along with four additional peaks at 2575 cm^−1^, 2473 cm^−1^, 1990 cm^−1^, and 1893 cm^−1^ labeled α, β, γ, and Δ, respectively. Also, the spectrum is approximately ten times stronger in intensity. This suggests that the deposited Eu film results in resonant Raman scattering when 532 nm excitation is used, possibly due to electronic transitions associated with Eu oxides and/or other compounds created by reaction with the EG. Our results are comparable to a report of EuO deposition on graphene show strong EuO Raman signatures with similar peak positions when using a 535 nm laser^40^.

Using the 455 nm probe, we acquire a Raman spectrum at a white spot in the Eu film, shown in Fig. 6d-iii. At this spot, the 2D and D peaks are particularly pronounced, the latter signifying the disruption of EG’s sp^2^ bonds. We propose that this is due to Eu-C bonding and possibly Eu and/or EuO intercalation. Eu and EuO films on graphene are known to intercalate under the graphene lattice, and can increase graphene conductivity, even inducing a band gap from the hybridization of EG/Eu/SiC buffer layer^38,39,54,63^.

Raman spectroscopic maps of the 2D peak position, G peak position, and D/G ratio of the graphene underneath the deposited Eu film are shown in Fig. 6e. We observe results similar to those discussed for the perimeter of the deposited Er film in Fig. 5, *except* the entire region under the deposited Eu is affected. For example, the 2D and G peak positions exhibit a red shift towards lower wavenumbers, see Fig. 6e-i,e-ii. The 2D peak position shifts approximately from 2748 ± 1 cm^−1^ in uncovered EG to 2715 ± 5 cm^−1^ for the deposited region. Likewise, the G peak shifts from 1595 ± 1 cm^−1^ in uncovered EG to 1577 ± 3 cm^−1^ for the deposited region. By contrast, studies of ultra-high vacuum deposition of EuO on CVD graphene result in a blue shift in the Raman spectrum^40^. Lastly, we find regions of substantially high D/G peak ratios in Eu covered EG, see Fig. 6e-iii. The spots in the spectroscopic map with ratios as high as 0.7 correlate to the white regions of the Eu film, akin to the spectrum in Fig. 6d-iii. It is clear that the EG at these spots is heavily defective.

As discussed for the case of deposited Er, as well as in the discussion in the paragraph above, the spectroscopic shifts in these peaks are consistent with damage to the graphene lattice, strain, oxidation, and intercalation.

**Figure 7:**
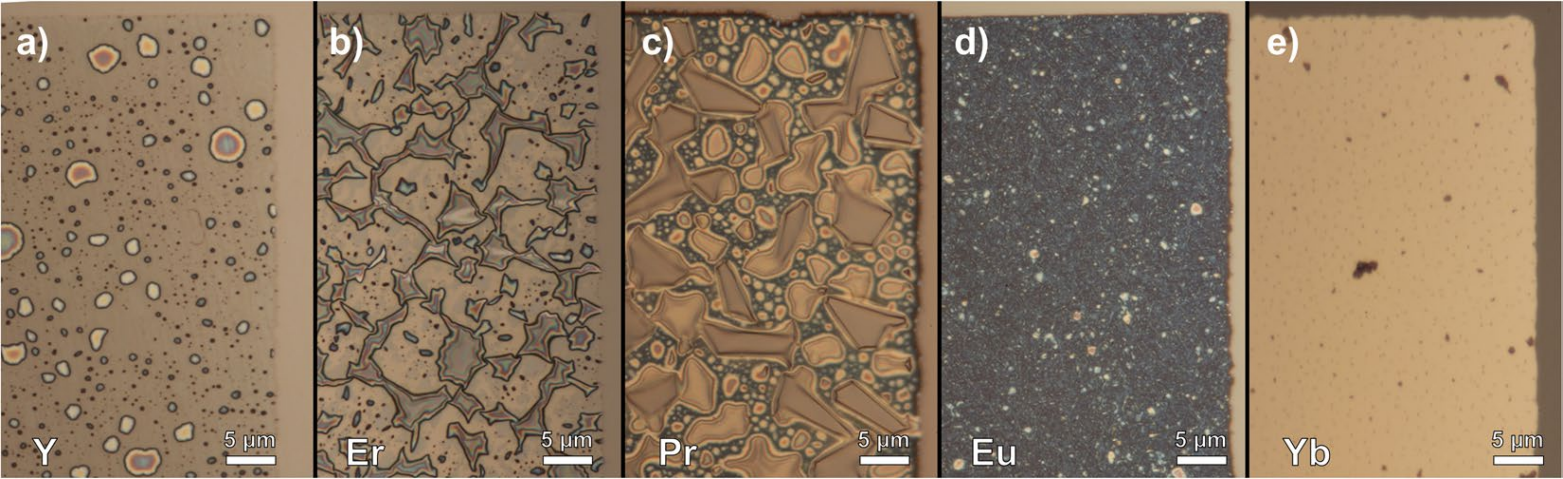
Optical images of (**a**) Y, (**b**) Er, (**c**) Pr, (**d**) Eu, and (**e**) Yb films 8 months after initial deposition. Y, Er, and Pr films show signs of adherence issues with substantially raised features. The Eu film has not changed at all, and Yb is mostly the same with the exception of minor pore formation.

The last of the rare earth metals, Yb, was comparatively unremarkable. Upon deposition, the film was relatively smooth with a surface roughness of only 5.0 ± 0.3 nm, see Table 1, a value second lowest in the low work function group where Y is the lowest: 2.7 ± 0.2 nm. However, evaluation of ambient-exposed deposited Yb over time proved it to be the most useful for contacts to EG contacts out of the five rare earth films in this study. Figure 7 shows optical images of the rare earth metal films deposited on EG taken 8 months after deposition. Y, Er, and Pr films, Fig. 7a–c respectively, show the formation of substantially tall irregular features, a progression of those observed shortly after deposition in Fig. 4. As discussed earlier, these features signify poor wetting and adhesion of the film to EG. For these films, the features were too large to measure with AFM without risk of tip damage. In contrast, the Eu film was nearly identical after 8 months, see Fig. 7d, having the same surface roughness as found initially, as shown in Table 1, signifying the various reactions as discussed above had already completed. The Yb film was most resilient to oxidation effects. After 8 months, the Yb film had a surface roughness of 4.8 ± 0.4 nm, within the uncertainty limits of the initial value shown in Table 1. However, after eight months these Yb films show the presence of irregular pores scattered throughout the film that extended down to the substrate, and Raman probing showed no indications of reactions unlike the previously discussed rare earth metals. Despite being the most favorable metal for processing, we still recommend capping Yb films with a subsequent deposition step, using for example, Ti/Au prior to removal from vacuum to slow oxidative effects for device applications.

**Figure 8:**
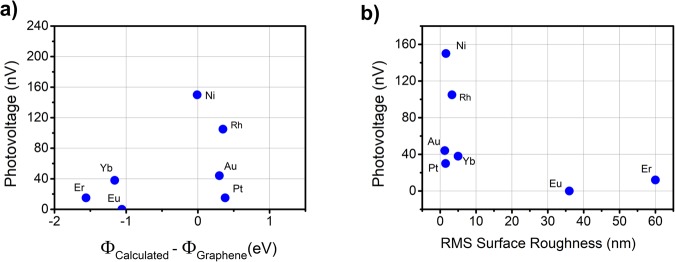
Maximum values for V_OC_ measured during photovoltage measurements with respect to the (**a**) difference in calculated work function with graphene and (**b**) surface roughness.

We measure the open circuit photovoltage, *V*_*OC*_, when the metal-EG interface is illuminated as a scanned 532 nm laser is moved across the pads; this approach is similar to other reports^21,25,26^. Figure 8a shows *V*_*OC*_ for the difference between the reported and calculated work functions for Eu, Yb, Er, Rh, Ni, Au and Pt, and Fig. 8b shows *V*_*OC*_ as a function of surface roughness. Since the metal-graphene perimeter interface can vary from sample to sample due to various effects (as exemplified in Figs 4, 6 and 7) such as oxidative reactions, the maximum V_OC_ is used in Fig. 8. We observe the largest signal at 150 nV from the Ni pads. Of the rare earth metals, the largest signal is from the Yb pads at 38 nV. Plotting the *V*_*OC*_ *vs*. the calculated work function difference with EG (Fig. 8a), no obvious correlation is observed, especially for the high work function metals which do not exhibit obvious oxidation effects. However, a plot of *V*_*OC*_ *vs*. surface roughness, see Fig. 8b, shows a more convincing relationship with a negative correlation between photovoltage and increasing surface roughness. This is not surprising because while the work function likely does influence the magnitude of a photovoltage response for the conditions employed in this work, its magnitude likely depends upon a continuous interface between the metal and graphene. For example, Yb had the strongest photovoltage response and was also the most robust rare earth metal film, see Fig. 7. For this reason, we identify Yb as a strong candidate for use as a low work function contact on graphene in graphene photonic devices.

## Conclusion

An analysis of eleven different metals deposited on EG shows that discrepancies exist between the measured and predicted work functions. On the one hand, we find that the work functions of the noble metals are similar to reported values, where the difference is due to impurities. On the other hand, the work functions of the rare earth metals vary greatly from prior work due to oxidation effects. For the as-deposited films, we observe no correlation between the photovoltage and metal work function for either noble metals or rare earth metals. There is some evidence that the metal-graphene interface integrity, indicated by the film roughness, may impact the photovoltage, especially in the case of rare earth metals. For low work function source or drain contacts on graphene, the most promising film is Yb. Of the rare earth films, Yb is one of the smoothest, gave the highest photovoltage response, and has the highest resistance to oxidation effects. Nevertheless, we recommend capping Yb films with Au or Ti/Au layer to retard oxidation and provide a good surface contact for probing.

## Methods

### Graphene Growth

Nominally monolayer (ML) epitaxial graphene (EG) was synthesized by Si sublimation, in a controlled Ar ambient, on nominally on-axis semi-insulating (0001) 6H-SiC using a commercial CVD reactor; additional details are found elsewhere^64–67^. All samples were synthesized under the same conditions using coupons from the same SiC substrate. With these synthesis conditions, Raman spectroscopy verified ML graphene was formed on the SiC terraces and 2 ML and sometimes 3 ML of graphene were formed on the steps. After graphene formation, samples were kept in a dry N_2_ box until metal deposition took place.

### Metal Deposition

Metals of varying work functions (see Table 1) were deposited on the graphene using electron-beam evaporation. The high work function metals group include Cr, Cu, Rh, Ni, Au, and Pt. The low work function (rare earth) group comprises Eu, Yb, Pr, Y, and Er. All of the electron-beam source material is greater than 99.99% pure. The background pressure of the evaporation chamber prior to deposition was between 1–3 × 10^−6^ Torr. Unless otherwise noted, 25–30 nm of metal was deposited. We used a shadow mask to avoid photoresist contamination for each deposition and the deposition rate was 2–3 Å s^−1^, with exceptions for Yb and Eu. For Yb and Eu, the deposition occurred at a rate much greater than 2–3 Å s^−1^ even at the lowest beam power due to the high evaporation rate of the source material. Three metals (two metals under test and Au acting as a control) were deposited on each graphene sample in overlapping but shifted patterns.

### Surface Probe Microscopy

The surface morphology and *Δϕ* of the graphene-metal and metal-metal interface were measured in the laboratory ambient using atomic force microscopy (AFM) and Kelvin probe force microscopy (KPFM) with a Bruker Dimension Icon. Bruker SPCM-PIT tips with a lift height of 90 nm were used and scans were 20–50 μm wide to prevent scanning interference effects at the graphene-metal film interface. The first scan was taken within ten minutes after removal from the evaporation chamber. The surface roughness (RMS) was extracted from AFM scans over areas of 10 μm × 10 μm; before metal deposition the graphene films had a roughness of 1.7 ± 0.2 nm. Optical images of the surface were taken with either the camera embedded in the Bruker AFM or an Olympus BX60M optical microscope. The temperature and humidity of the KPFM lab space were approximately 72 °C and 38% respectively.

### Spectroscopy

Photovoltage measurements were obtained by illuminating the sample with a 1 mW, 532 nm laser using a 1 μm spot size while recording the open circuit voltage measured by Be-doped Cu probe tips with a 50 μm spacing making contact with two identical deposited metal pads. A ThermoFisher DXRxi using a 9.6 mW, 532 nm or 6 mW, 455 nm confocal probe having a ~0.5 μm spot size was used to acquire the Raman spectroscopy data. X-ray Photoelectron Spectroscopy (XPS) using a Thermo Scientific K-Alpha system with a monochromatic Al source (Kα = 1486.6 eV) was used to analyze the chemical states of the rare earth metals. A low-energy electron flood gun was used for charge compensation and all core level peaks were fitted using a Shirley background subtraction.
